# Cultural differences in diagnosis and treatment perceptions: Turkish collectivistic representations of common mental disorders

**DOI:** 10.1080/17482631.2025.2459353

**Published:** 2025-01-28

**Authors:** Iclal Yildiz, Els Rommes, Enny Das

**Affiliations:** a Department of Gender & Diversity Studies, Radboud Social Cultural Research, Radboud University, Nijmegen, The Netherlands; b Department of Language and Communication, Centre for Language Studies, Radboud University, Nijmegen, The Netherlands

**Keywords:** Biomedical paradigm of health, ADHD, depression, collectivism, Turkish clients

## Abstract

**Purpose:**

Attention-deficit/hyperactivity disorder (ADHD) is less diagnosed among Turkish children, and Turkish clients drop out more often from depression treatments than Dutch clients. This article proposes that cultural differences in collectivistic versus individualistic perceptions of getting an ADHD diagnosis and being treated for depression might explain these ethnic disparities, which have been explored in this study.

**Methods:**

Nine focus group discussions with Turkish individuals and 18 interviews with primary mental health practitioners were conducted.

**Results:**

Findings show that Turkish participants do not view ADHD symptoms as problematic. Parents generally do not want children to be labelled as such and get professional help because they fear this will cause interpersonal problems. Depression is seen as problematic, as it disturbs social relationships and communities. However, Turkish participants prefer mediation to cognitive behavioural therapy, since the latter does not solve interpersonal problems.

**Conclusions:**

Our findings highlight a dissonance between individualistic/biomedical and collectivistic/socioecological views on health and wellbeing, in which the focus is on the individual for the Dutch versus the social group for the Turkish. To match Turkish clients’ needs, mental health professionals should tread carefully in ADHD labelling. With depression, professionals might consider interventions such as mediation in interpersonal conflicts to improve treatment adherence and outcomes.

## Introduction

The present research focuses on Turkish individuals in the Netherlands, one of the largest ethnic minority groups with approximately 431.000 inhabitants (de Mooij et al., 2022), and explores in which ways differences between Dutch professionals and their Turkish clients in individualism and collectivism influence perceptions of diagnosis and treatment of ADHD and depression, two of the most common mental disorders (World Health Organization, 2022). Literature suggests that attention-deficit/hyperactivity disorder (ADHD) is less often diagnosed among ethnic minority children, including Turkish children in the Netherlands, compared to Dutch children (Schmengler et al., 2021; Slobodin & Masalha, 2020; Zwirs et al., 2007). Furthermore, ethnic minority (Turkish) clients discontinue depression treatments more often than Dutch clients (de Wit et al., 2008; Teunissen et al., 2014), which suggests ethnic disparities in the diagnosis and treatment of mental disorders. These ethnic disparities in diagnosis and treatment can be explained by several factors. In this paper, we will focus on factors affecting the relationship between mental health professionals and clients, namely cultural differences in beliefs—i.e., illness and treatment representations—of ADHD and depression. The discussion of cultural differences is commonly linked with the application of cultural dimensions to analyse differences and variations between cultures. Scholars have identified and discussed many cultural dimensions, including individualism—collectivism, power distance, uncertainty avoidance, and femininity—masculinity, among others, that were popularized by Hofstede (1980, 2001). Although Hofstede’s work has been criticized, individualism-collectivism has received the most attention among these dimensions, and has thus been studied more extensively than other cultural dimensions (Fatehi et al., 2020). Similarly, this paper zooms in specifically on the individualism-collectivism (IND-COL) dimension. The IND-COL dimension refers to a different understanding of how individuals are embedded in social relationships, since cultures vary in how individuals in these cultures construct the self as independent or interdependent with their environment (Fatehi et al., 2020). The definition of individualism refers to the norms, values, and beliefs that individuals are born to be independent from their environment and will (or should) pursue values of autonomy, self-decision making, and personal benefits over a group’s benefits (Triandis, 1993). Thus, in individualistic societies, a person’s social behaviour is largely determined by personal goals. When a conflict arises between personal and group goals, it is considered acceptable to place goals, wishes and desires of the “self” ahead of collective ones. In collectivistic societies, social behaviour is determined largely by insisting on the interdependence of individuals and goals shared with some collective (such as “fitting in” and keeping intragroup harmony), and it is considered socially desirable to place collective goals ahead of personal goals (Triandis, 1989).

The IND-COL dimension is also reflected in how individuals view mental health problems, whether they are considered a(n individual) problem, and whether they should be solved individually (Kolstad & Gjesvik, 2014). In collectivistic cultures, symptoms are seen as problematic when they are a threat to the community, and the community is also considered responsible for solving mental health problems (Williams, 2003). In line with this, scholars have highlighted the importance of cultural mediation in illness meaning, which not only shapes (social) manifestations of distress in symptom reports and help-seeking but also influences the underlying psychophysiological processes that contribute to psychopathology and illness experience (Kirmayer & Bhugra, 2009). Such cultural mediations may influence Turkish perceptions on getting an ADHD diagnosis and being treated for depression, which may influence Turkish clients’ help-seeking behaviour and treatment adherence (Kirmayer & Bhugra, 2009). Conflicting values in individualism and collectivism have been cited as one of the primary barriers impending ethnic minority clients’ access to psychological help and treatment adherence, and are considered a key matter through which ethnic disparities in ADHD diagnosis and the treatment of depression are ameliorated (Armstrong & Swartzman, 2001). Addressing these conflicting values is key for improving practitioner–client relationships and reducing these disparities (Kilbourne et al., 2006). Indeed, scholars have emphasized the importance of several sociocultural factors in practitioner–client relationships that are key to the understanding and reduction of (mental) health disparities including ethnic disparities in the diagnosis and treatment of mental disorders. This prime area for (further) research has not been addressed in the literature so far (Amaro et al., 2021). This study’s focus on conflicting values in individualism and collectivism between Dutch mental health practitioners and Turkish clients contributes to filling this research gap, and helps clarify the sociocultural processes that shape and influence ethnic disparities in the diagnosis and treatment of mental disorders such as ADHD and depression. This will enable mental health professionals to better match Turkish clients’ needs.

Three types of mental disorders can be distinguished. First, neurodevelopmental disorders such as ADHD and autism, which is an umbrella term for neurologically based conditions that commonly appear early in childhood typically before school entry, and impair development of personal, social, academic, and/or occupational functioning. They typically involve difficulties with the acquisition, retention, or application of specific skills or sets of information and affect intellectual and cognitive ability, physical functioning, or both. Second, intellectual disabilities, which are considered a subcategory of neurodevelopmental disorders that does not include differences in functioning that are physical, referring to only those disorders characterized by limited mental capacity and difficulty with adaptive behaviours. Lastly, mental health disorders, which are generally defined as clinically significant disturbances in an individual’s cognition, emotion regulation, and behaviour, including conditions such as depression, anxiety disorders, and psychosis, among others. The main focus of this paper is on ADHD and depression, two of the most common mental disorders (World Health Organization, 2022). However, empirical findings of other mental disorders such as autism and intellectual disabilities are included where relevant.

The following research question guides the study:

What are Turkish collectivistic perceptions of diagnosis and treatment of common mental disorders, and in which ways are these different from what Dutch mental health professionals offer from an individualistic viewpoint?

## Conceptual framework

The biomedical model is the dominant model of health in Western mental healthcare settings. It assumes that disorders such as ADHD and depression are neurobiological dysfunctions, i.e., brain disorders. Symptoms are identified based on deviations from a behavioural norm (Deacon, 2013). The biomedical model is practiced in individualistic societies and based on ecological theory, which emphasizes the interaction of an organism (individual) and their biological environment (such as food, stress, etc.) (Germain, 1979). The biopsychosocial model of health expands on ecological theory and views mental disorders as an interplay between biological, psychological and social factors to understand interactions among biomedical and psychosocial causes of symptoms. Treatment is also linked to clients’ (social) environmental factors and their influence on behaviour. Nevertheless, diagnosis and treatment of ADHD and depression primarily follow the biomedical paradigm of health (Deacon, 2013; Erlandsson & Punzi, 2016), which is premised on individualism (Armstrong & Swartzman, 2001; Kirmayer, 2007; Kirmayer et al., 2018; Zeira, 2022). Mental health problems might have a social cause, but the individual has the autonomy to solve such problems (Lakeman, 2016; Viens, 2019).

Globally, the main intervention in mental health care in general and for depression in particular is cognitive behavioural therapy (CBT) (Hofmann et al., 2012; Terjesen et al., 2022). CBT explores the links between thoughts, emotions and behaviour, to enable a client to be aware of thoughts and emotions that influence their behaviour, and to address the problem that has caused a client’s depression (Fenn & Byrne, 2013). The assumption with CBT is that the factors that influence clients’ (health) behaviour are represented in the individual (Bracken & Thomas, 1999). Mental health problems are considered an individual problem, i.e., the solutions for mental health problems should come from the individual rather than the environment, because one has little control over changing the environment (Williams, 2003). However, such a view might be at odds with collectivistic illness and treatment representations (Armstrong & Swartzman, 2001). From a collectivistic perspective, the individual is interdependent with the community, and symptoms are seen as problematic when they are a threat to the community. Furthermore, not only the individual but the community is also considered responsible for solving mental health problems. This perspective thus guides the way individuals interpret mental health problems and think about solutions for those problems (Chigangaidze, 2021).

The socioecological model of health understands health to be affected by the interaction between the individual and its environment, which allows for a collectivistic orientation on illness and treatment (Ungar, 2002). This model of health is based on a combination of ecological and systems theory. Systems theory views mental disorders as a complex interaction between systems. It uses concepts of micro (individual), meso (interpersonal, organizational), and macro (community, policy, economic, political, socio-cultural) level interactions to assess how a disorder affects an individual. The assumption is that small perturbations at any level can have (negative) effects on clients’ overall functioning and well-being. Causes of symptoms may be attributed to factors from more than one level. These include, among others, the home environment, family relationships, social environment, school, work, socio-economic class, the larger community and society, and economic and political systems (Bronfenbrenner, 1977). Consequently, it drives interventions to holistically treat the whole system of influence on a client (Greene, 2017).

Solutions to address access to psychological help and reduce disparities in diagnosis and treatment are largely rooted in a deficit-based approach to treatment (addressing/closing the mental health treatment gap) (Kohn et al., 2004; Mathias et al., 2024; Prince et al., 2007). However, this approach overlooks the influence of the aforementioned factors influencing mental health conditions and recovery. Thus, many scholars have called for nuanced approaches to diagnosis and treatment that attend to these factors, and for community-based care that engage with lived experiences within the specific contexts of local (ethnic minority) communities in order to create space for interventions that build on community strengths and assets (Burgess et al., 2022). This invites fundamental changes in the ways in which mental health problems and their solutions may be conceptualized (Bemme, 2023). Such nuanced approaches may extend beyond neoliberal economic rationales focused on increasing access to biomedical care and treatment to address the treatment gap to a more expansive (collectivistic socioecological) logic of health and wellbeing that also includes assessments of how these factors, including overarching socio-cultural norms, play a role in shaping processes of and outcomes for mental health (Mathias et al., 2024).

## Methodology

This study is part of a previously published larger study on miscommunication between Turkish clients and Dutch professionals in (mental) health care (Yildiz et al., 2022), in which a different research question was answered. The current study involved a qualitative study using focus group discussions among Turkish individuals (*N* = 46), and semi-structured interviews among primary care practitioners (*N* = 18). These practitioners include a general practitioner (GP) and psychological wellbeing practitioners (PWPs, in Dutch: “praktijkondersteuners geestelijke gezondheidszorg/POH-GGZ”). Because of their gatekeeper role in Dutch specialized mental health care, these professionals are crucial in the diagnosis and treatment of ADHD and depression. GPs refer clients with ADHD and depressive symptoms to specialized care, where they can gain further assessment, diagnosis and if required, treatment. Clients with mild to moderate or uncomplicated forms of ADHD and depressive symptoms are referred to PWPs.

### Focus group discussions

Nine focus group discussions with 46 Turkish individuals took place between November 2019 and November 2022, each lasted between 60 and 120 minutes. The discussions were moderated by the first author, who is a second-generation bilingual Turkish researcher born in the Netherlands, and experienced in conducting qualitative research among Turkish individuals. The first author’s identity influenced her positionality in the following ways. The first author was viewed as an insider from the perspective of Turkish participants with respect to how Turkish participants view mental disorders and experience mental health care, but as an outsider from the perspective of Dutch professionals. This insider position could have made it difficult to maintain a critical distance to the data from Turkish participants, which is offset by discussing the data (analysis) with the co-authors who have a Dutch background. Regardless, we believe that being viewed as both an insider and outsider provides the first author an analytical advantage to investigate the issues that concern ethnic minority communities.

Participants were invited to discuss differences between Dutch and Turkish illness beliefs of depression and ADHD. A Turkish Master’s student fulfilled the role of observer and made field notes during and after the (first five) discussions. Depending on participants’ preferences, the discussions were conducted in Turkish or Dutch. Some became mixtures of both, whereas others were fully in Turkish. One focus group discussion was conducted through Zoom due to COVID-19 regulations. As the online discussion affected the flow of the discussion due to connection problems, a second Zoom discussion was conducted to cover all topics. Live discussions were generally held at places where participants came together for social or other purposes. The majority of the discussions took place in participants’ homes, except for a discussion that took place in a day care centre for Turkish elderly, and a discussion that took place in a mosque. The number of participants in each focus group discussion ranged from 3 to 9 participants, with a total of 46, representing diversity in terms of gender, age, migration generation and education level [see Table I]. The majority of the participants were parents who knew other Turkish parents who had children with ADHD (symptoms), or had a child with ADHD symptoms themselves. In the case of depression, the majority of the participants underwent or have had treatment for depressive symptoms themselves.

**Table I.** Focus group and participant characteristics.

Focus group	Number of participants
1a. Focus group characteristics	
1 2 3 4 5 6 7 8 9 Total	3 7 7 4 4 5 5 6 9 46
1b. Participant characteristics	
Number of participants Age range Gender (% male, female) Migration generation (% 1st, 2nd) Education level (% low, middle, high)^1^	46 20–81 13, 87 89, 11 60, 27, 13

### Interviews

Eighteen interviews took place between November 2019 and May 2022, with a GP and 17 psychological wellbeing practitioners. The interviews were conducted by the first author and two Dutch Master’s students of pedagogy and took approximately an hour. Due to COVID-19 regulations in effect during this time, the majority of the interviews were conducted online through video conferencing services, while a few of them took place offline. Interviewees were first asked to provide information on their education background and level of experience. Next, they were asked to describe differences between Dutch and Turkish clients’ beliefs about depression and ADHD. Follow-up questions were asked to deepen both interviews and focus group discussions, with a pilot tested topic list derived from Leventhal’s categories of illness representations (Leventhal et al., 1992) [Table II].

**Table II.** Topic list focus group discussions and interviews.

Topics/Categories	Description
Illness labelling	whether a particular behavior described in the DSM-V (e.g., for ADHD and depression) is considered problematic
Causes	factors or conditions believed to have caused the disorder
Solutions	the extent to which a disorder can be cured or controlled through particular treatment measures and behaviors, as well as whether a particular behavior described in the DSM-V needs psychological and/or psychiatric assistance

### Recruitment

Focus group participants and interviewees were face-to-face and online recruited through a combination of convenience and snowball sampling starting from existing networks of the first author and of the Turkish Master’s student. This meant that some of the focus group participants knew the moderator. This may have hindered their freedom to express themselves fully. However, the moderator saw that this turned out well and these pre-existing relationships prompted participants to open up and share their personal experiences and thoughts. This became evident from the fact that participants were eager to speak and had a lot to say, and the discussions required little moderation and few prompts. They talked in a we/them manner and were critical about the Dutch health care system and professionals. The first author recruited participants for the first (pilot) discussion through her network. Participants for the second, third, fourth, and fifth discussion were recruited through the Turkish Master’s student. For the sixth discussion and onwards, a few (Turkish) key persons from the network of the first author assisted in recruiting participants. The key persons were an acquaintance of the first author, two social workers, and a student, who informed people from their own networks about the study and invited them to participate in a focus group discussion for this study. Since the author, the Master’s student, and the key persons recruited participants already known to them, they reasonably assumed that participants would be able to inform important perspectives related to the topic of this study.

Nevertheless, the possibility of bias in the selection of focus group participants cannot be excluded. In addition, the majority of the participants were first-generation participants and had a low education level; however, this sample might be less representative of the general ethnic minority population in the Netherlands.

After being informed about the design and purpose of the study, all invited participants agreed to participate. Participants gave written consent through a consent form and could withdraw from participation at any time. They were compensated with a €10 gift voucher. In the recruitment of participants, we aimed for diversity in gender, age, generation, and education level. Participants born in Turkey were classified as first-generation, whereas participants born in the Netherlands were classified as second-generation Turkish individuals (Statistics Netherlands, n.d..). The ethics committee of Radboud Social Cultural Research approved the research methods and procedure. Thus, this research has received ethics approval from the research institute (Radboud Social Cultural Research) [code ECSW-2019-035].

Interviewees were recruited through several channels: 1) the first author’s network, 2) a Dutch annual PWP conference, in which a PWP approached the author with an interest to participate in the study, 3) through the network of one of the interviewed PWPs (snowball sampling), in which the interviewed PWP introduced the study to other PWPs, and 4) ten interviewees were recruited through several Dutch primary care umbrella organizations. The first author announced the study through the (offices of these) organizations and affiliated PWPs interested in participating in the study could contact the first author through email. To minimize potential biases in the selection of participants, we contacted primary care umbrella organizations with affiliated practices in areas with both large and small populations of ethnic minority clients. However, this might not exclude the possibility of bias. Participants found through this channel (also) self-selected if they wished to participate in the study. The disadvantage of self-selection is that mainly professionals who have affinity with the topic of this study might be interested to participate. Moreover, these professionals might (unconsciously) be more aware of cultural differences between the Turkish and Dutch population than other professionals.

Interviewees gave written consent through a consent form and could withdraw from participation at any time. They were compensated with a €10 gift voucher. Interviewees all had a Dutch (cultural) background, a ranging level of experience working in primary care (1–19 years) and diverse educational backgrounds, such as psychology, social work, and (psychiatric) nursing [Table III].

**Table III.** Interviewee characteristics.

Interviewee (pseudonym)	Ethnic background	Years of experience	Gender	Position	Education background
Stella Helena Toon Joke Marieke Monica Astrid Shirley Annelies Michelle Carmen Jennifer Gijs Inge Florian Vera Robert	Dutch Dutch Dutch Dutch Dutch Dutch Dutch Dutch Dutch Dutch Dutch Dutch Dutch Dutch Dutch Dutch Dutch	7 19 17 8 2–3 8 6 8 11 1 1 3 4 4 12 3 10	Female Female Male Female Female Female Female Female Female Female Female Female Male Female Male Female Male	PWP GP PWP PWP PWP PWP PWP PWP PWP PWP PWP PWP PWP PWP PWP PWP PWP	Pedagogics, social work Medicine, GP training Nursing, social work Creative therapy, PWP training Social work, PWP training Psychiatric nursing PWP training Social work, PWP training Psychiatric nursing, PWP training Social work, pedagogics Social work, PWP training Psychiatric nursing Nursing, PWP training Nursing, psychiatric nursing Social work, PWP training Psychiatric nursing, PWP training Psychology, PWP training

### Data analysis

The interviews and discussions were audio-recorded, transcribed and translated to English, participants were given pseudonyms, and data were analysed with Atlas.ti. A grounded theory inspired approach was adopted to analyse the data. The data were continuously categorized and compared, and themes were drawn out across discussions and interviews, and based on relevant literature, e.g., on ADHD and cognitive behavioural therapy. Transcripts were reviewed by the second author to ensure methodological rigour. To minimize the possibility of bias in data analysis, the first author discussed transcripts and analyses with the co-authors.

First, open coding has been performed regarding associations with/(collectivistic) representations of ADHD and depression, such as “stigma”, “access to help”, “effect on social relationships”, “(lack of) communication”, and “drop out”. Second, axial coding involved the identification of categories on a higher level, such as “ADHD symptoms and ADHD diagnosis”, “CBT and individual problem solving”, “CBT and lack of fit”. Finally, selective coding involved deriving the themes “ADHD causes interpersonal problems” and “depression disturbs communities and social relationships” from these categories. These themes answer the research question since they highlight general collectivistic representations of ADHD and depression, e.g., Turkish individuals’ focus on the social group rather than only on the individual when they make (mental) healthcare decisions (such as whether to seek help and adhere to treatment), due to viewing themselves and their environment as interdependent. Examples of quotes that illustrate these themes are Turkish participants who discussed that 1) among the Turkish, children’s stigmatization on the basis of psychiatric ADHD labels is a problem, because it might lead to children being less “marriageable” [FG8], and 2) Turkish parents often worry about whether their child will “sit still” during visits, because they fear visitors stigmatizing their child. For depression, examples of quotes are 1) a Turkish participant who suggested that a (Turkish) depressed person withdraws from the community, explaining that she has “not been hearing from [an acquaintance] for three weeks” [FG6], and participants who discussed that CBT does not work since “it is impossible to talk” [FG3] with the person they have a conflict with.

## Results

### ADHD

As mentioned in the introduction, ADHD is less often diagnosed among the Turkish than among the Dutch. Collectivistic perceptions of getting an ADHD diagnosis might explain this difference. For this reason, we explored Turkish collectivistic perceptions of getting an ADHD diagnosis. In general, participants and interviewees discussed that ADHD behaviour is not seen as (very) problematic by many Turkish parents for various reasons. Firstly, such a child is generally seen as “just busy”, which in Turkey is sometimes even valued among boys. According to focus group participants, “busy” boys are considered “*yırtıcı”* (ferocious) and “*gözü açık”* (shrewd), which are positively valued characteristics in boys. Furthermore, as a Turkish mother said, *“there is a culture of playing outside” [Aleyna, FG3]* among Turkish children, and - as a Dutch professional said - *“they [Turkish children] don’t have to sit in their rooms all the time.” [Annelies, PWP]*. Similarly, both professionals and parents feel that many Turkish parents are *“less strict about rules and having to do things [a certain way]” [Astrid, PWP]*. Consequently, parents and professionals believe that Turkish culture is more tolerant of busy, hyperactive behaviour than Dutch culture.

Secondly, ADHD behaviour is not seen as problematic by many Turkish parents because it does not hinder social relationships, which are considered crucial in collectivistic cultures. According to the parents we interviewed, children with ADHD symptoms can participate in social environments (e.g., socialize with other children). According to Turkish parents, this is different for a child with autism or intellectual disabilities who will have more trouble with social relationships. Consequently, the latter disorders are more quickly seen as problematic. Moreover, parenting a child with autism or an intellectual disability is deemed more taxing for parents, which hinders their ability to maintain their own social relationships.

Occasionally, Turkish parents do consider ADHD behaviour as a problem. When a child’s (busy) behaviour is too severe, it affects parents’ social relationships and/or may lead to problems at school. Both professionals and parents discussed that in these cases, *[parents say:] “maybe I should be stricter, maybe I should do this, do that” [Toon, PWP]* and try “all sorts of parenting interventions” to handle their children. Even though Turkish parents try various things, they generally do not want a child to get professional help and get labelled as they fear stigmatization of their child. Although stigmatization on the basis of psychological problems (labels) is nowadays much more acceptable among ethnic minority clients, which is also mentioned in the literature (Makowski & von Dem Knesebeck, 2017), according to focus group participants, this seems more of a problem if the diagnosed person is still a child. Our analysis indicates various reasons for this. First, as some of the participants mentioned, it might lead to children being “less marriageable”, and being marriageable is considered important in collectivistic cultures (Hofstede et al., 2010). As parents in some focus groups said: if a person *“went to the psychologist when little” [FG8]*, parents would not want their children to marry that person. Participants also mentioned that in the case of their child being diagnosed/having a label, (first-generation) Turkish parents often worry about whether their child will “sit still” during visits, because they fear visitors stigmatizing their child as someone who cannot sit still and will probably do damage in their house.

Other reasons have to do with Turkish individuals’ position in society. Many participants discussed that Turkish parents fear that their child will get an unjustified label because of stereotypical ideas by professionals. They explained that their children are more often perceived as “difficult” compared to Dutch pupils and their parenting is considered “less good” than parenting by Dutch parents. Furthermore, they fear that if their child gets a diagnostic label, this will result in being matched to a lower school level in the Dutch high school system or being transferred to special education. This fear is caused by the discrimination parents experience from schools. For instance, one of our focus group participants recalled that a school administrator told her that “foreigners”*“just become street rascals, not that they study” [Emine, FG8]*. Other participants also discussed that schools seem to think that Turkish parents are not interested in their child’s education. Parents thus believe that schools can use an ADHD label against them to affirm their view that Turkish children are not fit for education or that parents are not interested in their child’s education.

In which ways are Turkish perceptions and expectations of getting an ADHD diagnosis different from the Dutch? Many professionals and participants mentioned that as opposed to Turkish parents, Dutch parents generally view ADHD behaviour as problematic. According to professionals, Dutch parenting is “more structured” than Turkish parenting because Dutch parents are “adamant about rules” and children doing things a certain way. Consequently, Dutch parents *“quickly think that children do not fit a mold” [Monica, PWP]* and have a narrower range of what is perceived as normal behaviour.

As opposed to Turkish parents, Dutch parents do not seem to fear stigmatization of a labelled child as much. On the contrary, for some parents, a label means getting professional support. Consequently, parents are generally quick to *“come and say: I want an ADHD assessment” [Stella, PWP]*, and consult professionals to get a diagnosis.

This section shows how collectivistic value orientations influence Turkish perceptions of getting an ADHD diagnosis, and how this differs from Dutch perceptions. In summary, many Turkish parents do not consider ADHD behaviour as a problem because it does not hinder social relationships. Furthermore, they fear stigmatization of a labelled child. In contrast, Dutch parents are perceived to view ADHD behaviour as problematic and to have no issues with consulting professionals for a diagnosis, since a label grants children access to professional help.

## Depression

Whereas the Turkish view symptoms of ADHD as not (very) problematic, depressive symptoms are seen as problematic because they hinder interpersonal relationships. Nisa’s account illustrates how the Turkish experience depression: “*We have not been hearing from an acquaintance of mine for three weeks. They looked everywhere for him [.] because everyone thought something bad had happened. Eventually, he got in touch and said that he is depressed, that he doesn’t want to see anyone and that we shouldn’t contact him” [Nisa, FG6]*. Her account suggests that a depressed person withdraws from the community, and depression hinders interpersonal relationships and community participation.

According to Turkish participants, this is a problem for which help is needed. However, as mentioned in the introduction, Turkish clients drop out more often from depression treatments than Dutch clients, which suggests that Dutch treatment methods do not fit. To explain the reasons for drop out, we will explore how help for depression is realized in Turkey and how this contrasts with Dutch solutions for depression.

Participants mentioned that similar to the Netherlands, one of the main causes of depression in Turkey is problems in interpersonal relationships, i.e., conflicts between people. Other causes that were often mentioned were socio-economic difficulties and (post)migration (acculturation) challenges. Help for depression (in Turkey) is realized in various ways, according to participants. First, several participants recalled instances in which their spouse, family, friends and other acquaintances provided (emotional) support when they dealt with depression. They also encouraged others to look after or be there for someone who suffers from depression; for instance, a mother who tells her son to *“go call your [depressed] friend” [Selma, FG1]*. In Turkey, it is common that the community is involved in helping someone with depression, which is deemed a labour of love (in Turkish: *hatır meselesi*). The community signals that someone is not well, and mobilizes close relationships so the person can resume participation in the community.

Just as in the Netherlands, the Turkish seek support from loved ones (in Turkey) to deal with depression, as Murat discussed: “*I can sit with my parents [in Turkey] and vent. [.] I just vent about everything. [.] Here, [in the Netherlands] I don’t have someone to whom I can tell things. For example, things that happen in the family I can’t tell to you, to him, to him, I can’t tell that to someone” [Murat, FG5]*. Many professionals and participants similarly mentioned that the Turkish prefer to vent interpersonal problems to trusted loved ones (in Turkey) as a relief. Some wanted to vent to a psychologist, such as Melike: “*I went there [to the psychologist] to have some relief, to share my worries, but I came out being more burdened actually. I just got homework and was sent off [.]” [Melike, FG3]*. Melike’s experience suggests that she just wanted to vent about interpersonal problems but was given homework assignments instead.

Thirdly, depression needs to be solved through help in mediating conflicts, as Sami explained: *“Imagine I have a problem at home. [.] I can tell it to my parents, like: ‘I have an issue with X’. Then my mother can take X apart and say ‘I heard you two have issues’, she can give advice, there is potential to mediate” [Sami, FG5]*. Like Sami, several others mentioned that help for depression is realized through seeking a mediator in interpersonal conflicts. In Turkish culture, it is not common to directly talk with a person one has a conflict with. Others also mentioned that this is not a common thing to do: “*we Turkish people don’t come to a solution by talking [with eachother]” [Deniz, FG3]* and people are not going to confront the person they have a conflict with.

How do Turkish individuals’ collectivistic perceptions of solutions for depression differ from what Dutch professionals offer? As mentioned in the conceptual framework, the main intervention to treat depression is CBT that is focused on changing clients’ thoughts and behaviours, while in Turkey, the community interferes. Melike, who went to the psychologist with interpersonal problems, discussed that such treatment does not work: “*Every time when someone does something wrong [to me], I should talk with that person, and if I can’t talk, I should write a letter and send it [to that person]. [.] But it’s impossible to talk, it’s not the kind of person you can talk to” [Melike, FG3]*. Participants’ experiences are not unique to Dutch professionals, as they had the same experiences with Turkish psychologists. As we saw earlier, it is not an option for clients to talk directly to the person they have a conflict with. Professionals do not seem to be fully aware of this and do not support clients in interpersonal problems as they believe they should solve such problems themselves. Almost all interviewed professionals stated that the Turkish generally *“don’t take action” [Robert, PWP], “do something once and say it doesn’t work” [Annelies, PWP]* and generally do not follow up on their advices. For instance, professional Vera recalled her experience with a client who had a problem with her sister-in-law and recommended the client to express this to her, which she did not do: “*you give assertivity [training] and a lot of [advice] about how she can deal with this, but even when you just say: “look, just say that you feel uncomfortable”, she is not even able to make that step. [.] Whilst if she could express herself better, then she would feel better in such a situation” [Vera, PWP]*. All sorts of advices like Vera’s are provided during therapy, with the conviction that clients should directly talk to the person they have a conflict with. Clients seem to be (erroneously) judged as not assertive and not willing to “take action” during treatment. Treatment does not fit as the Turkish expect different treatment. From what they encounter in the community, they prefer a mediating role. GP Helena said that consequently, Turkish clients often drop out of treatment, which confirms the Dutch statistics on drop out by Turkish clients.

In summary, the Turkish (collectivistic) solution for depression lies in addressing interpersonal conflicts through community interference, venting to loved ones, and seeking a mediator without talking directly to the person people have a conflict with. In contrast, the Dutch solution is to seek professional help that is focused on changing individual clients’ behaviours, and talking directly with the person the client has a problem with. These differences in what is viewed as a fitting solution for depressive symptoms lead to clashes between Turkish clients and Dutch professionals. This is seen in how the Turkish reflect on treatment as making their symptoms worse and Dutch professionals’ negative judgements towards Turkish clients. As becomes clear from the analysis, such clashes can result in drop out from treatment.

## Discussion and conclusion

This study explored Turkish participants’ collectivistic perceptions of diagnosis and treatment of ADHD and depression, and in which ways these are different from what Dutch mental health professionals offer from an individualistic viewpoint. Our findings show that many Turkish parents do not consider ADHD behaviour of children as very problematic. When symptoms are considered somewhat problematic, parents do not want a label and professional help, mainly because they fear children’s stigmatization. Because of the discrimination that parents face by schools, they are even more vigilant to avoid a label.

A double dichotomization, of biology and culture, and of cultures (the difference presumption, which takes culture to explain cross-country differences in ADHD prevalence) is found in debates on the significance of the worldwide prevalence of ADHD (Pickering & Nie, 2016). Both biological and cultural differences may account for cross-country differences in ADHD prevalence. Regarding cultural differences, it is believed that ADHD may stem from sociocultural factors that are most common in American/Western societies (Faraone et al., 2003). The difference presumption appears in various guises, for example in the belief that the “West” and “East” have altogether different values, reflective of a number of dichotomies such as individualism and collectivism. This presumption has shaped cross-cultural debates (e.g., Conrad & Bergey, 2014; Faraone et al., 2003; Moffitt & Melchior, 2007; Timimi & Taylor, 2004) about the global rise of ADHD and whether ADHD is a valid medical diagnosis, as well as (ethical) debates about diagnosis and treatment in a particular (and potentially unhelpful) way. The question is whether differences in the rate of ADHD are a reflection of changes in its incidence or in society’s tolerance for behaviour that does not conform to expectations of “normal” behaviour. However, diagnostic labels play a key role in the allocation of resources to (specialized) mental health care. Only if ADHD is labelled as such are treatment regimens legitimated and remunerated, which is also the case in the Dutch mental healthcare context. In the Netherlands, DTCs (“Diagnosis Treatment Combination”) are used as the basis for remuneration negotiations between specialized mental health care and insurers (The Commonwealth Fund, 2020). Although this has not emerged from our data, Turkish parents and children might struggle with the management of symptoms. When a label is required for receiving relevant care, parents and children who might benefit from help are at risk of not receiving it. Furthermore, Turkish children who do have ADHD remain under the radar, get behind on academic milestones and do not receive adequate care.

In summary, our findings show that the traditional biomedical view of ADHD does not match all client groups alike. This means that professionals might want to take a wider (transcultural) orientation on the diagnosis (and treatment) of ADHD symptoms and should tread carefully with ADHD labels when Turkish parents are concerned with children’s stigmatization. Similar remarks have been made regarding other (North American ethnic minority) contexts as well (Na et al., 2016). Literature also suggests that stigma surrounding mental disorders, in this case psychiatric ADHD labels, might be more pronounced in collectivistic cultures than in individualistic cultures, due to (parental) concerns about maintaining social harmony in interpersonal relationships and preserving family reputation (Papadopoulos et al., 2013). Such stigma might influence help-seeking behaviour (Bathje & Pryor, 2011); hence, Turkish parents’ tendency to not seek help for ADHD symptoms because they fear children’s stigmatization and resulting disruptions in interpersonal relationships.

While ADHD symptoms are not regarded as very problematic by our focus group participants, the Turkish participants in our study do regard depressive symptoms as problematic since they hinder interpersonal relationships and community participation. However, they believe that cognition-based treatments like CBT treatment do not solve interpersonal conflicts, which leads to clashes with professionals and mutual misunderstanding. Our data suggest that Dutch (cognitive-behavioural) therapy aims to equip clients with tools to deal with interpersonal conflicts themselves, while clients seem to prefer mediation. Our findings are consistent with the literature, which suggests that collectivistic individuals in interpersonal conflict situations are primarily concerned with preserving harmonious relationships with others and avoiding direct confrontation in conflict situations (Hofstede, 2001; Li, 2006; Ohbuchi et al., 1999). Thus, such individuals may prefer non-direct methods of conflict resolution such as mediation instead (Brett, 2000; Hammer, 2005; Hofstede, 1989; Leung, 1997). Moreover, the Turkish people often deal with (postmigration) acculturation challenges such as a lack of familial support (from Turkey) (Laban et al., 2005). In Turkey, clients would presumably have family or trusted elders in their community mediate in interpersonal problems, but due to geographic distance, they might not be able to mediate. This means that Turkish clients are deprived of an important source of familial support. Our data indicates that amid this deprivation, depressive symptoms among the Turkish are addressed within the social group, reflecting a collectivistic orientation on coping strategies among the Turkish that also involves acquaintances in the community lending (or wanting to lend) (indirect) support to individuals. The focus is on adapting social structures and creating supportive environments to accommodate the individual in coping with depressive symptoms (Markus & Kitayama, 1991). This might contrast with individualistic cultures where perspectives on adequate coping might reflect an emphasis on individuals taking personal responsibility (Dehue, 2008). In conclusion, the (CBT) solution for depression does not match Turkish needs for a solution. To match their needs, professionals might explore options for mediation in interpersonal conflicts.

This study explored Turkish collectivistic perceptions of getting an ADHD diagnosis and of being treated for depression, and how this results in scepticism about the necessity of seeking an ADHD diagnosis and the effectiveness of depression treatments for people of Turkish descent living in the Netherlands. Our findings show that such perceptions primarily reflect differences in views on whether ADHD symptoms are problematic and in views on solutions for depression. This leads us to the conclusion that these differences highlight a dissonance between biomedical and socioecological views on health in which the focus is on the individual for the Dutch versus the social group for the Turkish, by whom ADHD and depressive symptoms are mainly interpreted in relation to the individual’s roles and functioning within the social group. Hence, mental health professionals might want to include socioecological perspectives on health when communicating about ADHD diagnoses and solutions for depression with Turkish clients, for which similar calls have been made in the global mental health literature (see Bemme & Kirmayer, 2020). Individual differences notwithstanding, professionals thus need to be aware of cultural differences in illness meaning, and understand that individualistic and collectivistic value orientations influence Dutch and Turkish differences in views on ADHD and in needs for solutions for depression. This will facilitate them to match Turkish needs, and in the case of depression, improve treatment adherence, outcomes and client satisfaction. Our findings might reflect similar (collectivistic) perceptions from other ethnic minority populations (across different countries), and of other mental disorders such as autism and intellectual disabilities. However, our findings reflect the perceptions of mostly first-generation Turkish individuals with low education levels, who might be less representative of the general (Turkish) ethnic minority population in the Netherlands. Future research might explore other ethnic (minority) groups with similar collectivistic value orientations living in a predominantly individualistic society, as well as compare Turkish communities in the Netherlands and Turkey in different contexts such as urban versus rural contexts. Communities residing in urban areas might be less subject to the mores and (collectivistic) value systems inherited from older generations/ancestors than communities residing in rural areas, where ancestors’ “social legacy” including mores and value systems might be more sustained. Future qualitative research might also further explore Turkish attitudes towards ADHD labelling. It is worthwhile to investigate why ADHD labelling is considered problematic when it concerns a child, and why an ADHD label is deemed stigmatizing while this is seemingly not the case for depression.

However, while our study highlights cultural differences in diagnosis and treatment perceptions, it is essential to guard against cultural essentialism, which oversimplifies and stereotypes ethnic groups. It is crucial to recognize that cultural practices and beliefs are dynamic and diverse within any ethnic group. We are well aware that not all individuals conform strictly to cultural norms, and there exists variability in their perceptions. Moreover, people negotiate and adapt their cultural practices in response to new environments and experiences, including within mental health care settings.

## Implications for policy and clinical practice

The policy and clinical implications of our findings are as follows. For ADHD, Turkish parents who (do) seek help for ADHD symptoms but are at risk of not receiving adequate help in specialized care in independent ADHD clinics, mental health organizations and hospitals can be supported through accessible (primary) care. Depending on the consequences a label has for Turkish children’s stigmatization, professionals might describe problematic behavioural patterns without labelling them as ADHD. For depression, referral schemes in (primary) care might facilitate Turkish clients’ engagement in interventions that could meet their needs for solutions for depression, so professionals can explore options for mediation in interpersonal conflicts.

## Data Availability

The data underlying this study are available upon reasonable request.

## Note

1. Low: primary or high school education, middle: vocational secondary education, high: higher vocational secondary or university-level education.
